# Silent Danger: Risk Factors and Outcomes of Fortuitously Discovered Uterine Rupture – A 41-Case Cohort Study

**DOI:** 10.12688/f1000research.164778.1

**Published:** 2025-06-13

**Authors:** Narjes karmous, Siwar Ghrab, Abdelwahab Masmoudi, Badreddine Bouguerra, Aymen Mabrouk, Anis ben Dhaou, Abdennour Karmous

**Affiliations:** 1 University of Tunis El Manar Faculty of Medicine of Tunis, Tunis, Tunisia; 2 Department B of Obstetrics and Gynecology, Charles Nicolle Hospital, Tinis, Tunisia; 3 General surgery department B, Charles Nicolle Hospital, Tunis, Tunisia; 4 Psychiatry Department, Razi Hospital, Manouba, Tunisia

**Keywords:** "Uterine rupture", "Silent uterine rupture", "Incidental diagnosis", "Scarred uterus", "Post-cesarean delivery complications", "Third-trimester obstetric emergencies".

## Abstract

**Background:**

Uterine rupture (UR) remains a major cause of maternal morbidity, especially in low-resource settings. While typically detected during labor, some cases are clinically silent, discovered incidentally during imaging/surgery, highlighting a knowledge gap in risk assessment. In Tunisia, 1.5% of pregnancies involve UR, mostly scar-related. The study aim was to identify factors associated with the development of complete UR in cases that were incidentally found during pregnancy or delivery.

**Methods:**

This was retrospective, longitudinal cohort study conducted over an eleven-year period, from January 2014 to December 2024, at the Gynaecology and Obstetrics department B, Charles Nicolle Hospital, Tunis, Tunisia. Asymptomatic UR cases (complete/incomplete) were analysed to compare clinical profiles, identify risk factors, and assess maternal and neonatal outcomes.

**Results:**

A total of 41 cases of asymptomatic UR were included, which accounted for an average of 50% of the UR cases. In a cohort comparing complete UR cases (N=27) and incomplete UR cases (N=14), significant differences in duration of pregnancy and labor were found. The mean gestational age was longer in the incomplete UR group (p=0.03), and the duration of labor was also significantly longer (p=0.006). No significant differences were observed in sociodemographic characteristics, quality of prenatal care, or complications such as gestational diabetes or preeclampsia. Nonsignificant factors included pregnancy interval, scars number and labor stagnation. The analysis showed two significant predictors of complete UR outcomes. Prolonged labor (>220 minutes) was strongly associated with increased odds of complete UR (OR=45.231, 95% CI=2.591-789.486, p=0.009) and lower maternal weight (<68 kg) correlated with reduced odds of incomplete UR (OR=0.033, 95% CI=0.001–0.837, p=0.039), suggesting a protective effect per kilogram decrease.

**Conclusion:**

Findings redefine UR as part of a broader clinical spectrum, not just an acute complication, enabling tailored surveillance and improved prevention in high-risk pregnancies.

## Introduction

Uterine rupture (UR) is still a significant cause of injury in the obstetrician’s field, it is particularly prevalent in low-income settings and contributes to the majority of maternal morbidity. ^1^ While typically detected during labor via classic symptoms, emerging evidence suggests that a subset of UR cases are likely to remain clinically silent. ^2^ These asymptomatic discoveries, which were made during imaging or surgery, represent a significant knowledge deficiency in the assessment of obstetric risks. ^3– 5^

In Tunisia, 1.5% of pregnancies have UR, the majority of which are caused by scarred uteri. Today, the diagnostic paradigm is primarily concerned with incidental presentations, which may or may not include silent cases that predispose to future obstetric issues. ^2^ Notably, the clinical importance of having complete or incomplete rupture in women that are symptomatic remains poorly understood, despite the potential difference in management and outcome.

This study analyzed a cohort of asymptomatic UR which includes both complete and incomplete UR, to address critical knowledge gaps regarding asymptomatic rupture. The analysis compared clinical and demographic characteristics of different UR types and identified specific risk factors for asymptomatic events. Particular attention was paid to the impact of evaluating the completeness of the UR on subsequent reproductive outcomes and to provide important data for parturient counseling and treatment.

Hence, the study aim was to identify factors associated with the development of complete UR in cases that were incidentally found during pregnancy or delivery.

## Methods

### Study design and setting

Retrospective, longitudinal and descriptive cohort study was conducted over an eleven-year period, from January 1, 2014, to December 31, 2024, at Gynaecology and Obstetrics department B, Charles Nicolle Hospital, Tunis, Tunisia.

### Study population

This retrospective case series analyzed asymptomatic parturients with incidentally discovered and intraoperatively confirmed complete UR.

The cohort was defined according to the following criteria:

▪ At least one previous uterine surgery (e.g., cesarean section, myomectomy);
▪ Incidental diagnosis of UR in a non-urgent clinical setting (e.g., routine antenatal imaging, elective cesarean section for other reasons, or non-urgent surgery);
▪ And complete clinical records, including surgical records, were available.

Symptomatic cases or UR discovered during labor or in an emergency setting were excluded to isolate the unique clinical features of asymptomatic, incidentally discovered ruptures.

### Variables

Data were retrospectively extracted from electronic medical records and focused on four domains:

▪ Maternal characteristics: Age, sociodemographic status, medical and surgical history, obstetric history …
▪ Characteristics of the pregnancy in question: Prenatal follow-up, delivery history …
▪ UR characteristics: Gestational age at diagnosis, clinical settings, anatomical type, surgical treatment …
▪ Maternal and neonatal outcomes: Maternal blood transfusion, intensive care unit (ICU) admission, neonatal status (Apgar score, birth weight, neonatal ICU admission …) …

### Statistical analysis

Data were entered and analysed with SPSS software (version 26.0, IBM Corp). Microsoft Office Excel was used to create the tables and graphs ( https://www.office.com/?omkt=fr-FR ).

For the primary analysis, we focused on the asymptomatic (randomized) group and compared cases of complete and incomplete UR. Bivariate comparisons were performed using the chi-square test or Fisher’s exact test for categorical variables and Student’s t test or Mann-Whitney U test for continuous variables.

Multivariate logistic regression models were then constructed to identify independent predictors of complete UR in incidentally diagnosed cases. Variables with a p value ≤ 0.20 in the univariate analysis were included in the model. Adjusted odds ratios (ORs) and 95% confidence intervals (CIs) were reported. A p value ≤ 0.05 was considered statistically significant. ^6, 7^

### Ethical considerations

The study protocol was approved on 13 February 2025 by the institutional ethics committee of Charles Nicolle Hospital, Tunis, Tunisia before conducting the study with approval number FWA 00032748- IORG0011243.

As this was a retrospective study using anonymized data, informed consent was waived.

## Results

During the study period, a total of 41 cases of asymptomatic UR were included.

**Figure 1.**
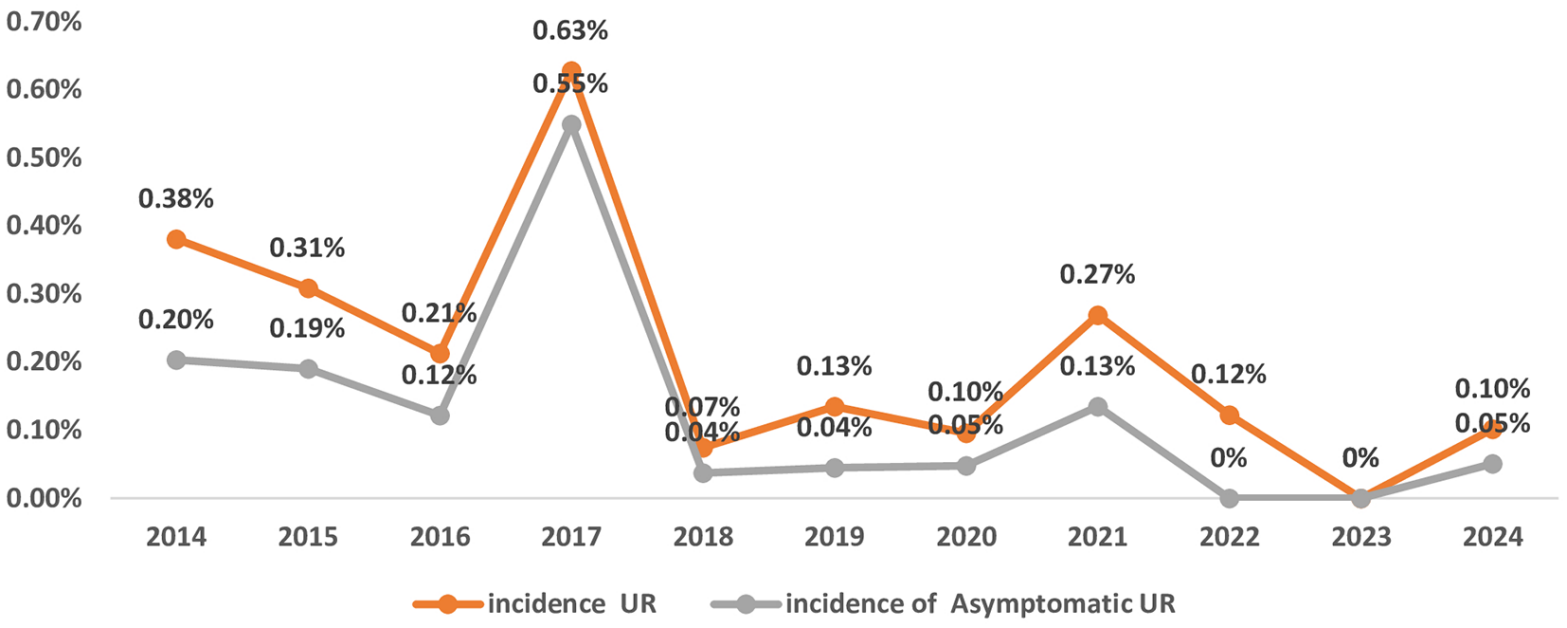
Trends in asymptomatic Uterine Rupture (UR) prevalence (2014-2024): proportion of asymptomatic cases among total UR at Charles Nicolle Hospital.

From 2014 to 2024 ( Table 1 and Figure 1), the maternity unit experienced a significant decrease in annual births, falling from 3939 to just 1964. UR cases peaked at 16 in 2017 but decreased afterward, with only one case reported in 2024. Cesarean deliveries reached their highest point in 2018 at 2033 but declined steadily to 1166 by 2024. In contrast, the number of vaginal deliveries remained relatively stable from 2019 onward, varying between 798 and 1043 each year. Asymptomatic UR accounted for an average of 50% of the UR cases over the eleven-year study period. It exhibited a fluctuating pattern, with the highest occurrence in 2017 (14 cases (88%).

**Table 1.** Annual distribution of asymptomatic uterine ruptures (UR), total of UR, and total births (2014–2024).

Year	Asymptomatic UR (N = 41)	Total UR (N = 69)	Cesarean deliveries (N = 15266)	Vaginal deliveries (N = 14600)	Total births (N = 29866)	Prevalence of Asymptomatic UR	Incidence of UR	Incidence of asymptomatic UR
2014	8	15	1547	2392	3939	53%	0,38%	0,20%
2015	8	13	1628	2582	4210	62%	0,31%	0,19%
2016	4	7	1289	2003	3292	57%	0,21%	0,12%
2017	14	16	1158	1390	2548	88%	0,63%	0,55%
2018	1	2	2033	654	2687	50%	0,07%	0,04%
2019	1	3	1340	892	2232	33%	0,13%	0,04%
2020	1	2	1215	870	2085	50%	0,10%	0,05%
2021	3	6	1223	1006	2229	50%	0,27%	0,13%
2022	0	3	1411	1043	2454	0%	0,12%	0%
2023	0	0	1256	970	2226	0%	0%	0%
2024	1	2	1166	798	1964	50%	0,10%	0,05%

The average age was 33.29 ± 4.9 years (24-44 years). Among the 41 parturients, 51% were classified as having an average socioeconomic status, followed by 32% with a high status, and 17% with a low status. In terms of educational attainment, 49% had completed secondary education, 32% held a university-level degree, and 19% had attained only primary education.

Table 2 presents descriptive statistics for various variables related to the study population including weight, height, gravidity, parity, term, interpregnancy interval, and duration of labor.

**Table 2.** Descriptive statistics of parturients demographics and labor parameters.

Variable	Median	Minimum	25 ^th^ percentile (Q1)	75 ^th^ percentile (Q3)	Maximum
Weight (kg)	72	61	69	75	80
Height (m)	1.61	1.53	1.57	1.66	1.73
Gravidity	3	1	2	4	5
Parity	3	2	2	3.5	6
Term (weeks)	39	22	37	39	41
Interpregnancy Interval (months)	24	6	12	48	72
Duration of Labor (min)	170	60	63.75	300	600

Thirty-six (88%) of the parturients arrived in active labor. Clinically, seven parturients (17%) developed hypertension during pregnancy, and four parturients (10%) were diagnosed with gestational diabetes Labor stagnation occurred in 7 individuals (17%).

Regarding the time of UR diagnosis, 40 parturients (98%) were diagnosed after delivery, whilst 1 parturient (2%) was diagnosed during labor.

Table 3 presents UR type (complete or incomplete), maternal and neonatal outcomes.

**Table 3.** Uterine Rupture (UR) type, maternal and neonatal outcomes.

Variable	Statistics
Complete UR N (%)	27 (66%)
Incomplete UR N (%)	14 (34%)
Transfusion N (%)	7 (17%)
Urological Injury N (%)	1 (2%)
Duration of Hospitalization (days) median [IQR]	3 [2-7]
APGAR Score at 5 Minutes median [IQR]	10 [6-10]
Birth Weight (PFN) median [IQR]	3460 [1150-4050]
Neonatal Hospitalization N (%)	1 (2%)
Neonatal Death N (%)	0

In a cohort comparing complete UR cases (N = 27) and incomplete UR cases (N = 14) ( Table 4), significant differences in duration of pregnancy and labor were found. The mean gestational age was longer in the incomplete UR group (38.86 weeks vs. 36.85 weeks, p = 0.03), and the duration of labor was also significantly longer (305.45 minutes vs. 142.94 minutes, p = 0.006). Trends showed that the parity and the proportion of parturients with multiple scars was higher in the complete UR group (3.22 vs. 2.50, p = 0.071 and 82% vs. 18%, p = 0.092 respectively). No significant differences were observed in demographic characteristics (age, weight, BMI), socioeconomic status or education level, quality of prenatal care, or complications such as gestational diabetes or preeclampsia. Preeclampsia occurred only in the incomplete UR group (14% vs. 0%, p = 0.111). Nonsignificant factors included pregnancy interval, number of scars and labor stagnation.

**Table 4.** Univariate analysis comparing complete Uterine Ruptures (UR) cases and incomplete UR cases.

Characteristics	Complete UR (N = 27)	Incomplete UR (N = 14)	P
**Age (Mean ± SD)**	33 ± 5	34 ± 6	0,634
**Weight (kg)**	72 ± 3	70 ± 5	0,115
**Height (m)**	1.62 ± 0.06	1.60 ± 0.04	0,246
**Body mass index**	27.51 ± 1.59	27.17 ± 1.20	0,422
**Gravidity**	3.30 ± 1.17	3.07 ± 1.21	0,523
**Parity**	3.22 ± 1.37	2.50 ± 0.94	0,071
**Gestational age (weeks)**	36.85 ± 3.98	38.86 ± 1.70	**0,03**
**Scar number**	1.52 ± 0.75	1.36 ± 0.74	0,333
One scar	13 (54%)	11 (46%)	0,092
More than one scar	14 (82%)	3 (18%)	
**Socioeconomic status**			0,922
- Poor	4 (15%)	3 (21%)	
- Average	14 (52%)	7 (50%)	
- Good	9 (33%)	4 (29%)	
**Education Level**			0,744
- Primary	5 (19%)	3 (21%)	
- Secondary	13 (48%)	7 (50%)	
- University	9 (33%)	4 (29%)	
**Prenatal Follow-up **			0,901
- Poor	2 (7%)	1 (7%)	
- Average	11 (41%)	6 (43%)	
- Good	14 (52%)	7 (50%)	
**Gestational Diabetes**	2 (50%)	2 (50%)	1
**Pre-eclampsia **	0 (0%)	2 (14%)	0,111
**Stagnation of Dilation**	5 (19%)	2 (14%)	0,385
**Moment of Discovery**			1
- During Labor	1 (4%)	0 (0%)	
- After Delivery	26 (96%)	14 (100%)	
**Interval (months)**	30.92 ± 17.26	28.71 ± 20.87	0,592
**Duration of Labor (min)**	142.94 ± 111.78	305.45 ± 150.76	**0,006**

The analysis identified two significant predictors of complete UR outcomes ( Table 5): Prolonged labor (>220 minutes) was strongly associated with increased odds of complete UR (OR = 45.231, 95% CI = 2.591-789.486, p = 0.009), indicating a 45-fold higher risk compared to shorter labor durations and lower maternal weight (<68 kg) correlated with reduced odds of incomplete UR (OR = 0.033, 95% CI = 0.001–0.837, p = 0.039), suggesting a protective effect per kilogram decrease.

**Table 5.** Multivariate analysis for identification of predictors of complete Uterine Rupture (UR) outcomes.

	p	OR	Confidence interval	
			Low	High
Parity	0,132	0,318	0,072	1,413
Number of scar	0,113	10,343	0,574	186,467
Prolonged labor (>220 minutes)	0,009	45,231	2,591	789,486
Weight <68 kg	0,039	0,033	0,001	0,837

## Discussion

Between 2014 and 2024, the number of deliveries per year at the institution decreased by 53% (from 3939 to 1964), and the number of cesarean sections decreased by 43% (from a peak of 2033 in 2018 to 1166 in 2024). These trends likely reflect changes in medical practices and demographic factors over the decade. The high proportion of asymptomatic UR (median 50% of cases) highlights the limitations of symptom-based diagnosis, especially in years with increasing prevalence (up to 88%).

At the same time, the number of UR cases decreased from 16 in 2017 to 1 in 2024. This is consistent with the literature linking prior cesarean sections to the risk of UR. ^8, 9^ This suggests that the decline in surgical births and improvements in surgical techniques may have led to the reduction in UR. Of note, there was also a decrease in asymptomatic cases of UR after 2018 (from 14 in 2017 to 1-3 per year), which may be due to advances in prenatal imaging, as described by Giampaolino et al. ^10^ However, 98% of UR cases in this cohort were diagnosed after delivery, which contradicts studies advocating prenatal MRI/ultrasound for early detection, indicating a major gap in prenatal surveillance. ^11, 12^

This cohort showed no demographic (age, body mass index, socioeconomic status) or obstetric (quality of antenatal care, gestational diabetes) associations with UR outcomes. However, two new predictors emerged:

Prolonged labor: Labor longer than 220 minutes increased the odds of complete UR by 45 times (OR=45.231, p=0.009), which is consistent with Savukyne et al., ^9^ who identified prolonged labor as a major risk factor.

Lower maternal weight: Weight less than 68 kg decreased the odds of incomplete UR (OR=0.033/kg, p=0.039), a finding that contradicts the literature that frequently links obesity to obstetric risk. This warrants further investigation of biomechanical or metabolic protective mechanisms.

Gestational age (38.86 weeks vs. 36.85 weeks, p=0.03) and delivery time (306 minutes vs. 143 minutes, p=0.006) were also longer in incomplete UR cases, suggesting that sustained uterine pressure may have led to partial UR.

Despite advances in imaging technology, 98% of UR cases were diagnosed after birth. This highlights the underutilization of prenatal MRI and ultrasound, an important tool for detecting scar dehiscence or placenta accreta. ^13, 14^ Although the treatment of UR has not been described in detail, the dramatic decline in cases may be due to improved surgical interventions ^14^ or conservative strategies for asymptomatic cases. ^10^

### Study strengths and limitations

This study’s analysis of UR trends over a decade (2014–2024) offers significant contributions to the literature, particularly through its identification of novel risk factors- prolonged labor (>220 minutes) and lower maternal weight- and its demonstration of a 94% decline in UR cases coinciding with reduced cesarean delivery rates, reflecting broader improvements in obstetric practices. The cohort of 41 UR cases, larger than most prior studies, ^8^ enabled robust comparisons between complete and incomplete UR types, revealing key differences in gestational age and labor duration.

However, the retrospective, single-center design introduces limitations, including potential selection bias and underpowered subgroup analyses (e.g., preeclampsia rates), while the near-exclusive reliance on post-delivery diagnosis (98% of cases) contrasts sharply with literature advocating prenatal MRI/ultrasound for early detection of scar dehiscence or placental anomalies. ^11, 12^

### Recommendations

The findings advance UR risk stratification by expanding beyond traditional predictors (prior uterine surgery) and highlight the need for prospective, multicenter studies to validate novel associations while integrating advanced imaging and standardized management protocols to bridge diagnostic and care disparities observed across institutions.

## Conclusions

Our findings redefine UR as part of the clinical picture rather than simply an acute complication of obstetrics. This paradigm shift enables customized surveillance of the scarred uterus and improves detection and prevention capabilities in high-risk obstetrics.

## Ethical considerations

We confirm that we have read the Journal’s position on issues involved in ethical publication and affirm that this report is consistent with those guidelines.

The study protocol was approved on 13 February 2025 by the institutional ethics committee of Charles Nicolle Hospital, Tunis, Tunisia before conducting the study (approval number: FWA 00032748- IORG0011243).

## Consent to participate

As this was a retrospective study using anonymized data, informed consent was waived.

## Data Availability

All data sets can be assessed and all study findings reported in the article are shared via Harvard Dataverse: “Silent Danger: Risk Factors and Outcomes of Fortuitously Discovered Uterine Rupture – A 41-Case Cohort Study”, https://doi.org/10.7910/DVN/D9OO16. ^15^ This project contains the following:
• Dataset silent UR- English • Study findings silent UR Harvard Dataverse: “Silent Danger: Risk Factors and Outcomes of Fortuitously Discovered Uterine Rupture – A 41-Case Cohort Study”, https://doi.org/10.7910/DVN/D9OO16. ^15^ This project contains the following: Questionnaire (in English) This work has been reported in line with the STROBE guidelines. ^16^ Harvard Dataverse: “Silent Danger: Risk Factors and Outcomes of Fortuitously Discovered Uterine Rupture – A 41-Case Cohort Study”, https://doi.org/10.7910/DVN/D9OO16. ^15^ This project contains the following: STROBE Checklist Data are available under the terms of the Creative Commons Zero “No rights reserved” data waiver (CC0 1.0 Public domain dedication).
