# Silent Danger: Risk Factors and Outcomes of Fortuitously Discovered Uterine Rupture – A 41-Case Cohort Study

**DOI:** 10.12688/f1000research.164778.3

**Published:** 2025-08-28

**Authors:** Narjes karmous, Siwar Ghrab, Abdelwahab Masmoudi, Badreddine Bouguerra, Aymen Mabrouk, Anis ben Dhaou, Abdennour Karmous

**Affiliations:** 1 Department B of Obstetrics and Gynecology, Charles Nicolle Hospital, Tinis, Tunisia; 2 University of Tunis El Manar Faculty of Medicine of Tunis, Tunis, Tunisia; 3 General surgery department B, Charles Nicolle Hospital, Tunis, Tunisia; 4 Psychiatry Department, Razi Hospital, Manouba, Tunisia

**Keywords:** "Uterine rupture", "Silent uterine rupture", "Incidental diagnosis", "Scarred uterus", "Post-cesarean delivery complications", "Third-trimester obstetric emergencies".

## Abstract

**Background:**

Uterine rupture (UR) remains a major cause of maternal morbidity, especially in low-resource settings. While typically detected during labor, some cases are clinically silent, discovered incidentally during imaging/surgery, highlighting a knowledge gap in risk assessment. In Tunisia, 1.5% of pregnancies involve UR, mostly scar-related. The study aim was to identify factors associated with the development of fortuitously discovered UR in cases that were incidentally found during pregnancy or delivery.

**Methods:**

This was retrospective, longitudinal cohort study conducted over an eleven-year period, from January 2014 to December 2024, at the Gynaecology and Obstetrics department B, Charles Nicolle Hospital, Tunis, Tunisia. Asymptomatic UR cases (complete/incomplete) were analysed to compare clinical profiles, identify risk factors, and assess maternal and neonatal outcomes.

**Results:**

A total of 41 cases of asymptomatic UR were included, which accounted for an average of 50% of the UR cases. In a cohort comparing complete UR cases (N=27) and incomplete UR cases (N=14), significant differences in duration of pregnancy and labor were found. The mean gestational age was longer in the incomplete UR group (p=0.03), and the duration of labor was also significantly longer (p=0.006). No significant differences were observed in sociodemographic characteristics, quality of prenatal care, or complications such as gestational diabetes or preeclampsia. Nonsignificant factors included pregnancy interval, scars number and labor stagnation. The analysis showed two significant predictors of complete UR outcomes. Prolonged labor (>220 minutes) was strongly associated with increased odds of complete UR (OR=45.231, 95% CI=2.591-789.486, p=0.009) and lower maternal weight (<68 kg) correlated with reduced odds of incomplete UR (OR=0.033, 95% CI=0.001–0.837, p=0.039), suggesting a protective effect per kilogram maternal body weight decrease.

**Conclusion:**

Findings redefine UR as part of a broader clinical spectrum, not just an acute obstetric complication. Early identification of associated risk factors such as prolonged labor and maternal weight could inform targeted surveillance in high-risk pregnancies.

## Introduction

Uterine rupture (UR) is still a significant cause of injury in the obstetrician’s field, it is particularly prevalent in low-income settings and contributes to the majority of maternal morbidity. ^1^ While typically detected during labor via classic symptoms, emerging evidence suggests that a subset of UR cases are likely to remain clinically silent. ^2^ These asymptomatic discoveries, which were made during imaging or surgery, represent a significant knowledge deficiency in the assessment of obstetric risks. ^3– 5^

Although most URs occur in previously scarred uteri, rare etiologies have been reported in unscarred uteri, including uterine anomalies, connective tissue disorders, or placenta accreta spectrum disorders such as placenta percreta. ^6^

In developing countries, several studies have addressed silent or symptomatic UR. For example, Ebeigbe et al. (Nigeria), ^7^ Kadowa (Uganda) ^8^ and Fofie & Baffoe (Ghana) ^9^ documented how healthcare access, sociocultural factors, and surgical history affect UR incidence.

In Tunisia, 1.5% of pregnancies have UR, the majority of which are caused by scarred uteri. Today, the diagnostic paradigm is primarily concerned with incidental presentations, which may or may not include silent cases that predispose to future obstetric issues. ^2^ Notably, the clinical importance of having complete or incomplete UR in women that are symptomatic remains poorly understood, despite the potential difference in management and outcome.

This study analyzed a cohort of asymptomatic UR which includes both complete and incomplete UR, to address critical knowledge gaps regarding asymptomatic rupture. The analysis compared clinical and demographic characteristics of different UR types and identified specific risk factors for asymptomatic events. Particular attention was paid to the impact of evaluating the completeness of the UR on subsequent reproductive outcomes and to provide important data for parturient counseling and treatment.

Hence, the study aim was to identify factors associated with the development of UR in cases that were fortuitously discovered during pregnancy or delivery in asymptomatic women.

## Methods

### Study design and setting

Retrospective, longitudinal and descriptive cohort study was conducted over an eleven-year period, from January 1, 2014, to December 31, 2024, at Gynaecology and Obstetrics department B, Charles Nicolle Hospital, Tunis, Tunisia.

### Study population

This study included asymptomatic pregnant women with incidentally discovered and intraoperatively confirmed complete UR.

The cohort was defined according to the following criteria:

**Inclusion criteria**

▪ Intraoperative or postpartum diagnosis of UR in the absence of any preceding clinical signs or symptoms (e.g., abdominal pain, vaginal bleeding, or signs of fetal distress).
▪ Complete and available medical and surgical records.

**Exclusion criteria**

▪ Cases in which UR was suspected preoperatively, before delivery based on clinical symptoms or abnormal findings during labor monitoring
▪ Incomplete or missing data.

**Definitions**

Two types of UR, complete and incomplete, were distinguished based on whether the overlying serosa of the uterus was involved: ^10, 11^

▪ A complete UR was defined as a full-thickness disruption involving the entire uterine wall, including the serosa.
▪ An incomplete UR was defined as a partial myometrial defect, often contained by the serosa or peritoneum, with no communication with the peritoneal cavity.

**Figure 1.**
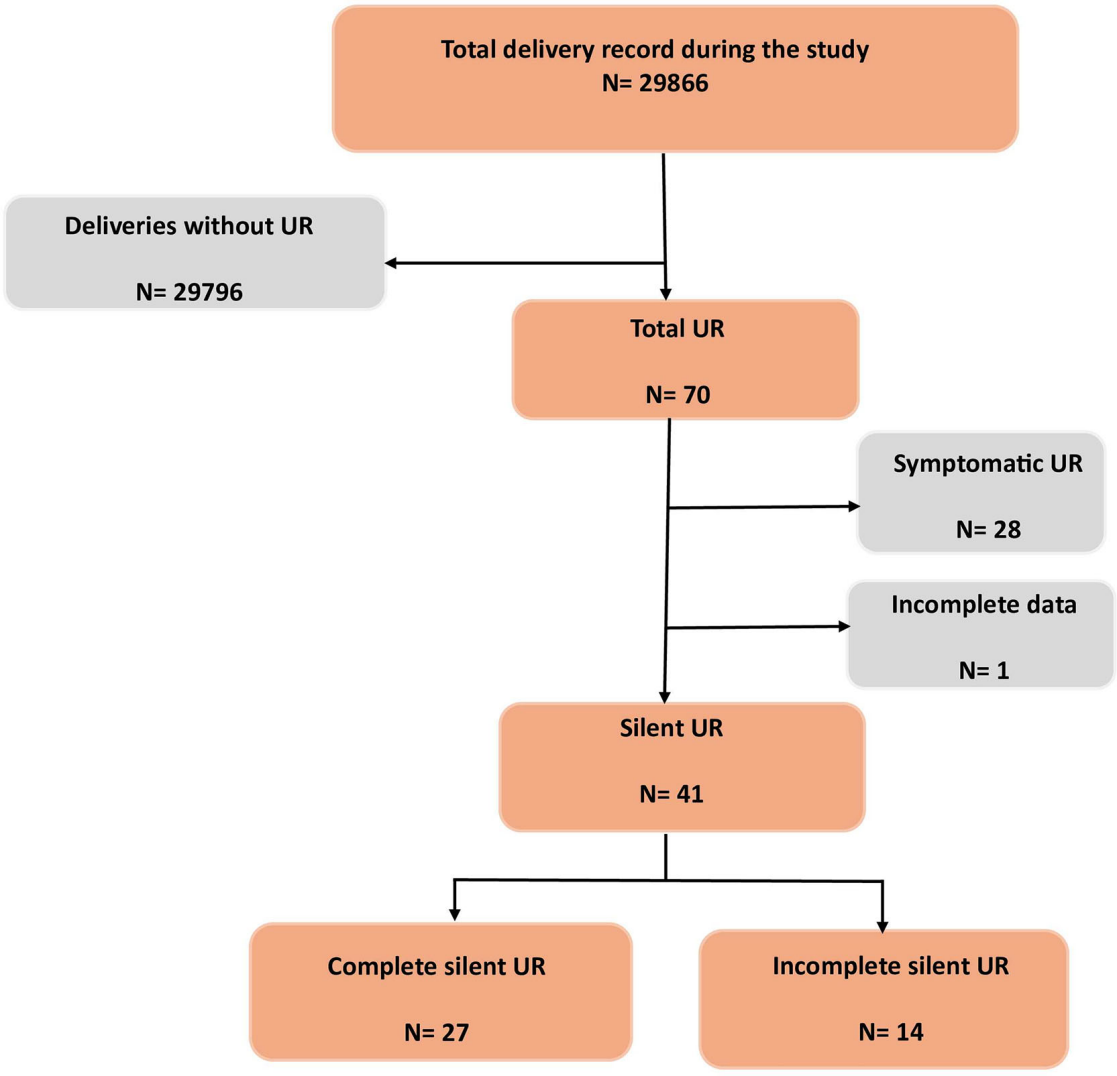
Flowchart of the study population.

A study flowchart detailing case selection and exclusions has been developed ( Figure 1).

### Variables

Data were retrospectively extracted from electronic medical records and focused on four domains:

▪ Maternal characteristics: Age, Body mass index (BMI), socioeconomic status, educational level, medical and surgical history, obstetric history …
▪ Characteristics of the pregnancy and delivery in question: Prenatal follow-up, uterine scar number, interpregnancy interval, mode and timing of delivery …
▪ UR characteristics: Gestational age at diagnosis, clinical settings, type of rupture (complete vs incomplete), associated intraoperative findings, surgical treatment …
▪ Maternal and neonatal outcomes: Maternal blood transfusion, intensive care unit (ICU) admission, neonatal status (Apgar score, birth weight, neonatal ICU admission …) …

### Statistical analysis

Data were entered and analysed with SPSS software (version 26.0, IBM Corp). Microsoft Office Excel was used to create the tables and graphs ( https://www.office.com/?omkt=fr-FR ).

For comparative analysis, complete UR versus incomplete UR cases were assessed using the chi-square test or Fisher’s exact test for categorical variables and Student’s t test or Mann-Whitney U test for continuous variables.

Multivariate logistic regression models were then constructed to identify independent predictors of complete UR in incidentally diagnosed cases. Variables with a p value ≤ 0.20 in the univariate analysis were included in the model. Adjusted odds ratios (ORs) and 95% confidence intervals (CIs) were reported. A p value ≤ 0.05 was considered statistically significant. ^12, 13^

Given the small sample size -particularly in the incomplete UR group- CI ranges were expected to be wide, and this limitation was considered in the interpretation of results.

### Ethical considerations

The study protocol was approved on 13 February 2025 by the institutional ethics committee of Charles Nicolle Hospital, Tunis, Tunisia before conducting the study with approval number FWA 00032748- IORG0011243.

As this was a retrospective study using anonymized data, informed consent was waived.

## Results

During the study period, a total of 41 cases of silent UR were included.

**Figure 2.**
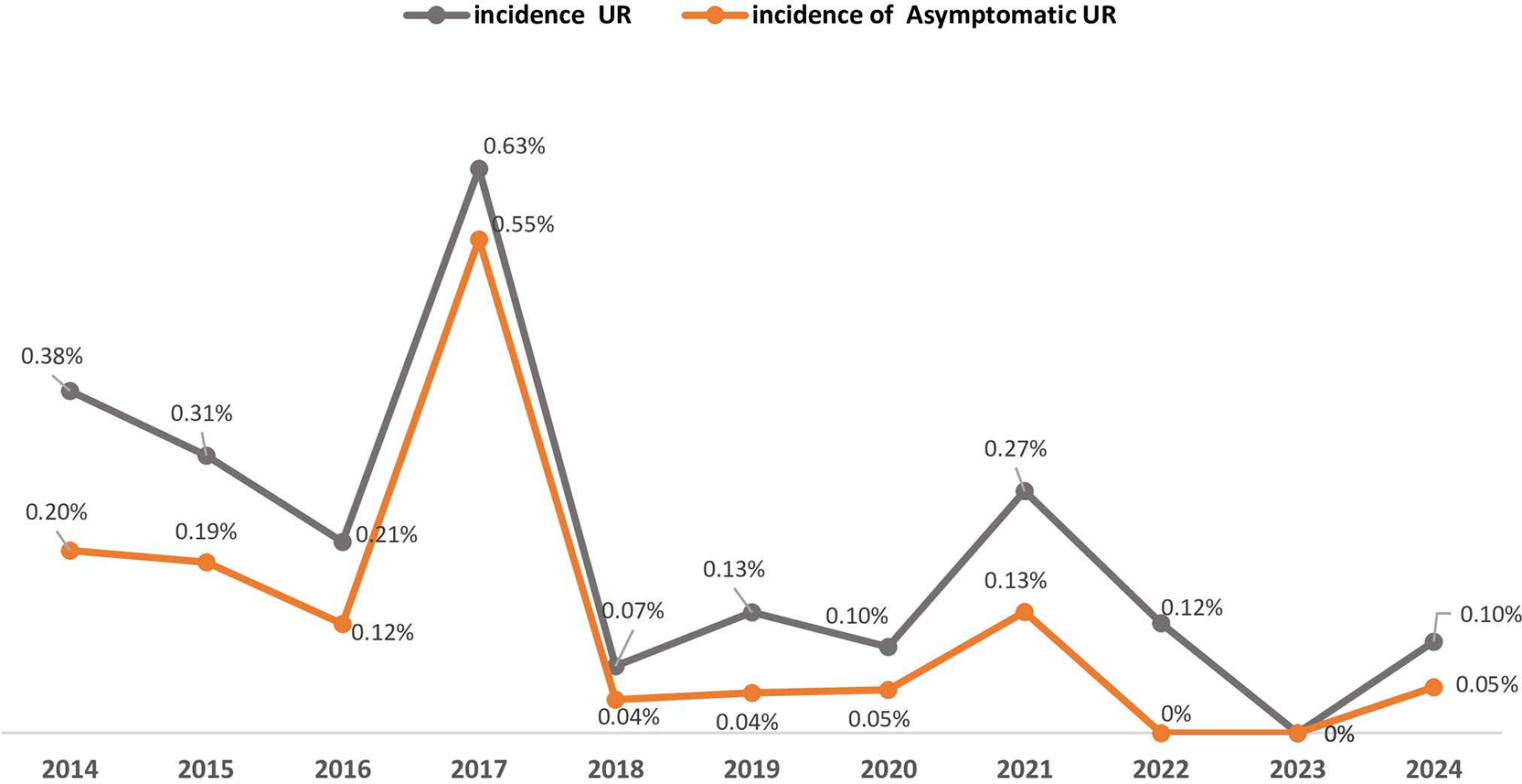
Trends in asymptomatic Uterine Rupture (UR) prevalence (2014-2024): proportion of asymptomatic cases among total UR at Charles Nicolle Hospital.

From 2014 to 2024 ( Table 1 and Figure 2), the maternity unit experienced a significant decrease in annual births, falling from 3939 to just 1964. UR cases peaked at 16 in 2017 but decreased afterward, with only one case reported in 2024. Cesarean deliveries reached their highest point in 2018 at 2033 but declined steadily to 1166 by 2024. In contrast, the number of vaginal deliveries remained relatively stable from 2019 onward, varying between 798 and 1043 each year. Asymptomatic UR accounted for an average of 50% of the UR cases over the eleven-year study period. It exhibited a fluctuating pattern, with the highest occurrence in 2017 (14 cases (88%).

**Table 1.** Annual distribution of asymptomatic uterine ruptures (UR), total of UR, and total births (2014–2024).

Year	Asymptomatic UR (N = 41)	Total UR (N = 69)	Cesarean deliveries (N = 15266)	Vaginal deliveries (N = 14600)	Total births (N = 29866)	Prevalence of Asymptomatic UR	Incidence of UR	Incidence of asymptomatic UR
2014	8	15	1547	2392	3939	53%	0,38%	0,20%
2015	8	13	1628	2582	4210	62%	0,31%	0,19%
2016	4	7	1289	2003	3292	57%	0,21%	0,12%
2017	14	16	1158	1390	2548	88%	0,63%	0,55%
2018	1	2	2033	654	2687	50%	0,07%	0,04%
2019	1	3	1340	892	2232	33%	0,13%	0,04%
2020	1	2	1215	870	2085	50%	0,10%	0,05%
2021	3	6	1223	1006	2229	50%	0,27%	0,13%
2022	0	3	1411	1043	2454	0%	0,12%	0%
2023	0	0	1256	970	2226	0%	0%	0%
2024	1	2	1166	798	1964	50%	0,10%	0,05%

The average age was 33.29 ± 4.9 years (24-44 years). Among the 41 women with silent UR, 51% were classified as having an average socioeconomic status, followed by 32% with a high status, and 17% with a low status. In terms of educational attainment, 49% had completed secondary education, 32% held a university-level degree, and 19% had attained only primary education.

Table 2 presents descriptive statistics for various variables related to the study population including BMI, gravidity, parity, term, interpregnancy interval, and duration of labor.

**Table 2.** Descriptive statistics of parturients demographics and labor parameters.

Variable	Median	Minimum	25 ^th^ percentile (Q1)	75 ^th^ percentile (Q3)	Maximum
Body mass index (kg/m ^2^)	27.61	25.30	26.40	28.48	30.75
Gravidity	3	1	2	4	5
Parity	3	2	2	3.5	6
Term (weeks)	39	22	37	39	41
Interpregnancy Interval (months)	24	6	12	48	72
Duration of Labor (min)	170	60	63.75	300	600

Clinically, seven women with silent UR (17%) developed hypertension during pregnancy, and four (10%) were diagnosed with gestational diabetes.

Thirty-six (88%) of the study population arrived in active labor. Labor stagnation occurred in 7 individuals (17%).

Regarding the time of UR diagnosis, 40 URs (98%) were diagnosed after delivery, whilst one (2%) was diagnosed during labor.

Table 3 presents UR type (complete or incomplete), maternal and neonatal outcomes.

**Table 3.** Uterine Rupture (UR) type, maternal and neonatal outcomes.

Variable	Statistics
Complete UR N (%)	27 (66%)
Incomplete UR N (%)	14 (34%)
Transfusion N (%)	7 (17%)
Maternal estimated blood loss (ml) median [IQR]	350 [250-400]
Packed red cell transfused N median [IQR]	0 [0-0]
Urological Injury N (%)	1 (2%)
Duration of Hospitalization (days) median [IQR]	3 [2-7]
APGAR Score at 5 Minutes median [IQR]	10 [6-10]
Birth Weight (PFN) median [IQR]	3460 [1150-4050]
Neonatal Hospitalization N (%)	1 (2%)
Neonatal Death N (%)	0

### Comparison between complete and incomplete silent UR

In a cohort comparing complete silent UR cases (N = 27) and incomplete silent UR cases (N = 14) ( Table 4), significant differences in duration of pregnancy and labor were found. The mean gestational age was longer in the incomplete UR group (38.86 weeks vs. 36.85 weeks, p = 0.03), and the duration of labor was also significantly longer (305.45 minutes vs. 142.94 minutes, p = 0.006). Trends showed that the parity and the proportion of parturients with multiple scars was higher in the complete UR group (3.22 vs. 2.50, p = 0.071 and 82% vs. 18%, p = 0.092 respectively). No significant differences were observed in demographic characteristics (age, BMI), socioeconomic status or education level, quality of prenatal care, or complications such as gestational diabetes or preeclampsia. Preeclampsia occurred only in the incomplete UR group (14% vs. 0%, p = 0.111). Nonsignificant factors included pregnancy interval, number of scars and labor stagnation.

**Table 4.** Univariate analysis comparing complete Uterine Ruptures (UR) cases and incomplete UR cases.

Characteristics	Complete UR (N = 27)	Incomplete UR (N = 14)	P
**Age (Mean ± SD)**	33 ± 5	34 ± 6	0,634
**Body mass index**	27.51 ± 1.59	27.17 ± 1.20	0,422
**Gravidity**	3.30 ± 1.17	3.07 ± 1.21	0,523
**Parity**	3.22 ± 1.37	2.50 ± 0.94	0,071
**Gestational age (weeks)**	36.85 ± 3.98	38.86 ± 1.70	**0,03**
**Scar number**	1.52 ± 0.75	1.36 ± 0.74	0,333
One scar	13 (54%)	11 (46%)	0,092
More than one scar	14 (82%)	3 (18%)	
**Socioeconomic status**			0,922
- Poor	4 (15%)	3 (21%)	
- Average	14 (52%)	7 (50%)	
- Good	9 (33%)	4 (29%)	
**Education Level**			0,744
- Primary	5 (19%)	3 (21%)	
- Secondary	13 (48%)	7 (50%)	
- University	9 (33%)	4 (29%)	
**Prenatal Follow-up **			0,901
- Poor	2 (7%)	1 (7%)	
- Average	11 (41%)	6 (43%)	
- Good	14 (52%)	7 (50%)	
**Gestational Diabetes**	2 (50%)	2 (50%)	1
**Pre-eclampsia **	0 (0%)	2 (14%)	0,111
**Stagnation of Dilation**	5 (19%)	2 (14%)	0,385
**Moment of Discovery**			1
- During Labor	1 (4%)	0 (0%)	
- After Delivery	26 (96%)	14 (100%)	
**Interval (months)**	30.92 ± 17.26	28.71 ± 20.87	0,592
**Duration of Labor (min)**	142.94 ± 111.78	305.45 ± 150.76	**0,006**

### Multivariate analysis

Table 5 shows the logistic regression model for predictors of complete silent UR. Two significant predictors were identified:

**Prolonged labor (>220 minutes)** was strongly associated with increased odds of complete silent UR (OR = 45.231, 95% CI = 2.591-789.486, p = 0.009), indicating a 45-fold higher risk compared to shorter labor durations.

**Lower maternal weight (<68 kg)** correlated with reduced odds of incomplete silent UR (OR = 0.033, 95% CI = 0.001–0.837, p = 0.039), suggesting a protective effect per kilogram maternal body weight decrease.

**Table 5.** Multivariate analysis for identification of predictors of complete Uterine Rupture (UR) outcomes.

	p	OR	Confidence interval	
			Low	High
Parity	0,132	0,318	0,072	1,413
Number of scar	0,113	10,343	0,574	186,467
Prolonged labor (>220 minutes)	0,009	45,231	2,591	789,486
Weight <68 kg	0,039	0,033	0,001	0,837

Only 7 women (17%) in the cohort had a weight < 68 kg and 14 women had incomplete UR, which likely contributed to the wide CI in the multivariate analysis.

## Discussion

Between 2014 and 2024, the number of deliveries per year at the institution decreased by 53% (from 3939 to 1964), and the number of cesarean sections decreased by 43% (from a peak of 2033 in 2018 to 1166 in 2024).

At the same time, the number of UR cases decreased from 16 in 2017 to 1 in 2024. Of note, there was also a decrease in asymptomatic cases of UR after 2018 (from 14 in 2017 to 1-3 per year) ( Figure 2).

This sustained decline is likely multifactorial. Several key factors may have contributed ^14– 16^:

▪ **Declining cesarean rates:** The reduction in the number of cesarean deliveries led to a smaller population of women with uterine scars. Fewer uterine scars meant fewer potential weak points that could undergo asymptomatic dehiscence in subsequent pregnancies, thus lowering the number of silent UR cases.
▪ **Improved prevention strategies and monitoring:** Enhanced antenatal surveillance and stricter labor management protocols, including closer monitoring of uterine scar integrity, likely reduced the occurrence of silent UR. Timely interventions could prevent small dehiscences from progressing into complete ruptures.
▪ **More cautious Trial of labor after cesarean (TOLAC) practices:** TOLAC may have been undertaken with stricter selection criteria and more rigorous monitoring (continuous fetal heart rate tracing, careful labor progress assessment), minimizing the likelihood of unnoticed scar separation.
▪ **Earlier recognition and management:** Better training and awareness among clinicians, combined with greater availability of intraoperative uterine inspection during repeat cesareans and early use of imaging when scar complications were suspected, likely enabled earlier detection and management of scar weaknesses before they evolved into silent ruptures.

Although the majority of URs occurred in women with previous cesarean sections, rare etiologies such as placenta accreta spectrum, particularly placenta percreta, have been reported even in unscarred uteri. ^6^ Awareness of these uncommon causes remains essential.

Other causes of intra-abdominal hemorrhage during pregnancy should be kept in mind, including placental abruption, UR in unscarred uterus, ruptured splenic or hepatic aneurysms. ^17^ The etiologies of spontaneous hemoperitoneum in pregnancy are rarely established with the spontaneous rupture of proliferative vasculature relates to some unknown risk factors such as endometriosis, adenomyosis and a relevant history of ovarian tumor removal. ^18^

Our findings showed that 98% of silent URs were diagnosed post-delivery, suggesting a potential role for enhanced antenatal imaging. High-resolution ultrasound and magnetic resonance imaging (MRI) can detect thinning of uterine scars or dehiscence, but their use remains limited in daily practice. Alalaf et al. demonstrated that during the first stage of labor, a lower uterine segment (LUS) thickness ≤ 2.3 mm and myometrial thickness ≤ 1.9 mm are significantly associated with uterine defects including dehiscence. ^19^ The most recent systematic review and meta-analysis suggests that an LUS > 3.65 mm should be safe for a TOLAC, 2–3.65 mm is probably safe, and <2 mm identifies a patient at higher risk for UR/dehiscence. ^20^

This cohort showed no demographic (age, body mass index, socioeconomic status) or obstetric (quality of antenatal care, gestational diabetes) associations with UR outcomes. However, two new predictors emerged:

**Prolonged labor:** Labor longer than 220 minutes increased the odds of complete UR by 45 times (OR=45.231, p=0.009), which is consistent with Savukyne et al., ^15^ who identified prolonged labor as a major risk factor.

**Lower maternal weight:** Weight less than 68 kg decreased the odds of incomplete UR (OR=0.033/kg, p=0.039). Several large cohort studies have shown that lower maternal weight is associated with a significantly reduced risk of UR during TOLAC. For example, Cahill et al. reported that women weighing less than 68 kg had a 70% lower risk of UR compared to heavier women (adjusted OR = 0.3; 95% CI: 0.1–0.8), even after adjusting for birth weight. ^21^ Similarly, Bujold et al. found that women with a BMI < 25 kg/m ^2^ were significantly less likely to experience rupture compared to those with BMI > 30 kg/m ^2^, the latter showing a 2.3-fold increased risk (OR = 2.3; 95% CI: 1.4–3.8). ^22^

Obesity may predispose to rupture through multiple mechanisms: impaired wound healing due to chronic inflammation and proinflammatory cytokines, poor collagen remodeling, and the formation of fibrotic, poorly vascularized scars. In addition, Landon et al. identified obesity (BMI > 30 kg/m ^2^) as an independent risk factor for UR (OR = 2.7; 95% CI: 1.1–6.6), regardless of fetal size. ^23^ While macrosomia (>4000 g) is also associated with an increased risk of rupture (OR = 2.5; 95% CI: 1.3–4.8 per Jastrow et al.), ^24^ maternal obesity appears to exert an even stronger effect. Grobman et al. found that both variables independently predicted UR, but maternal BMI had a greater impact than neonatal weight in multivariable models. ^25^ Moreover, Chauhan et al. highlighted that maternal obesity contributes to dysfunctional labor patterns, delayed diagnosis due to increased adiposity, and reduced uterine contractility, all of which increase the cumulative risk of scar failure. ^26^

Together, these findings suggest that maternal weight and body composition are key determinants of uterine scar performance during labor and should be included in individualized TOLAC counseling and decision-making.

Gestational age (38.86 weeks vs. 36.85 weeks, p=0.03) and delivery time (306 minutes vs. 143 minutes, p=0.006) were also longer in incomplete UR cases, suggesting that sustained uterine pressure may have led to partial UR.

Monitoring tools also warrant attention:

▪ **Cardiotocography (CTG)** abnormalities such as persistent bradycardia or late decelerations can be early indicators of UR. ^27^
▪ **Changes in fetal head position** during labor may signal uterine dehiscence, particularly when associated with cephalopelvic disproportion or arrest of descent. ^28^

Although no cases of pulmonary embolism were reported in our series, thromboprophylaxis during and after cesarean delivery is essential, particularly in women experiencing severe hemorrhage. ^29^

### Study strengths and limitations

This study’s analysis of UR trends over a decade (2014–2024) offers significant contributions to the literature, particularly through:

▪ Its identification of novel risk factors: prolonged labor (>220 minutes) was strongly associated with increased odds of complete silent UR and lower maternal weight (<68 kg) was correlated with reduced odds of incomplete silent UR suggesting a protective effect per kilogram maternal body weight decrease.
▪ Its demonstration of a 94% decline in UR cases coinciding with reduced cesarean delivery rates, reflecting broader improvements in obstetric practices.
▪ The cohort of 41 UR cases was larger than most prior studies. ^14^

However, the retrospective, single-center design introduces limitations, including potential selection bias and underpowered subgroup analyses (e.g., preeclampsia rates), while the near-exclusive reliance on post-delivery diagnosis (98% of cases) contrasts sharply with literature advocating prenatal MRI/ultrasound for early detection of scar dehiscence or placental anomalies. ^30, 31^

### Implications

▪ **Enhanced prenatal imaging** including systematic assessment of LUS thickness via ultrasound or MRI may facilitate early identification of scar defects in high-risk women, particularly those with multiple previous cesareans.
▪ **Continuous intrapartum monitoring** (CTG) should be emphasized during labor, as it may help detect subtle fetal signs suggestive of dehiscence or UR.
▪ **Women with prolonged labor, multiple uterine scars, or low LUS thickness** require closer surveillance, as they may have an elevated risk of complete UR even in the absence of clinical symptoms.
▪ **Individualized antenatal counseling** and **planned delivery strategies**, taking into account maternal weight, fetal size, and obstetric history, may reduce the risk of missed silent ruptures and improve both maternal and neonatal outcomes.
▪ **Maternal weight** may serve as an indirect marker of UR risk. In our study, lower maternal weight (< 68 kg) was independently associated with a reduced risk of incomplete UR. While this finding requires confirmation in larger cohorts, it suggests that maternal anthropometric factors could be integrated into future predictive models for safer TOLAC planning.

### Recommendations

The study’s strengths lie in its focus on a rarely investigated presentation of UR and its identification of novel risk factors. Two independent predictors of complete silent UR emerged: prolonged labor duration (>220 minutes) and maternal weight greater than 68 kg. However, given the limited sample size and retrospective design, these associations should be interpreted with caution. Future prospective, multicenter studies are warranted to validate these predictors and explore their utility in clinical decision-making for TOLAC.

## Conclusions

This study highlights that UR can occur without clinical signs and may remain undetected until surgical or postpartum evaluation. Among women with silent UR, we identified two independent predictors of complete rupture: prolonged labor duration > 220 minutes and maternal weight < 68 kg. These findings may contribute to improved risk stratification in pregnant women undergoing trial of labor after caesarean.

## Ethical considerations

We confirm that we have read the Journal’s position on issues involved in ethical publication and affirm that this report is consistent with those guidelines.

The study protocol was approved on 13 February 2025 by the institutional ethics committee of Charles Nicolle Hospital, Tunis, Tunisia before conducting the study (approval number: FWA 00032748- IORG0011243).

## Consent to participate

As this was a retrospective study using anonymized data, informed consent was waived.

## Data Availability

All data sets can be assessed and all study findings reported in the article are shared via Harvard Dataverse: “Silent Danger: Risk Factors and Outcomes of Fortuitously Discovered Uterine Rupture – A 41-Case Cohort Study”, https://doi.org/10.7910/DVN/D9OO16. ^32^ This project contains the following:
• Dataset silent UR- English. • Study findings silent UR. Harvard Dataverse: “Silent Danger: Risk Factors and Outcomes of Fortuitously Discovered Uterine Rupture – A 41-Case Cohort Study”, https://doi.org/10.7910/DVN/D9OO16. ^32^ This project contains the following: Questionnaire (in English). This work has been reported in line with the STROBE guidelines. ^33^ Harvard Dataverse: “Silent Danger: Risk Factors and Outcomes of Fortuitously Discovered Uterine Rupture – A 41-Case Cohort Study”, https://doi.org/10.7910/DVN/D9OO16. ^32^ This project contains the following: STROBE Checklist Data are available under the terms of the Creative Commons Zero “No rights reserved” data waiver (CC0 1.0 Public domain dedication).
