# Supplementary material for: Silent Danger: Risk Factors and Outcomes of Fortuitously Discovered Uterine Rupture – A 41-Case Cohort Study
Source: F1000Res. 2025 Aug 28;14:585. Originally published 2025 Jun 13. [Version 3] doi: 10.12688/f1000research.164778.3 (PMC12395129; doi:10.12688/f1000research.164778.3)
Supplement: Peer review history

# Reviewer report: Reviewer response for version 2

**DOI:** 10.5256/f1000research.186053.r403829

**Published:** 2025-08-28

**Authors:** Phuc Nhon Nguyen

**Affiliations:** 1 Tu Du Hospital, Ho Chi Minh City, Vietnam

Thank you for your revision.

 The paper is more improved in scientific points.

Is the work clearly and accurately presented and does it cite the current literature?

Partly

If applicable, is the statistical analysis and its interpretation appropriate?

I cannot comment. A qualified statistician is required.

Are all the source data underlying the results available to ensure full reproducibility?

Partly

Is the study design appropriate and is the work technically sound?

Partly

Are the conclusions drawn adequately supported by the results?

Partly

Are sufficient details of methods and analysis provided to allow replication by others?

Partly

Reviewer Expertise:

Pregnancy pathology

I confirm that I have read this submission and believe that I have an appropriate level of expertise to confirm that it is of an acceptable scientific standard.

# Reviewer report: Reviewer response for version 2

**DOI:** 10.5256/f1000research.186053.r403828

**Published:** 2025-08-28

**Authors:** Mathew Olumide Adebisi

**Affiliations:** 1 Obstetrics and Gynaecology, Federal Teaching Hospital Ido-Ekiti (Ringgold ID: 605037), Ido Ekiti, Ekiti, Nigeria; 2 Afe Babalola University, Ado-Ekiti, Ekiti, Nigeria

No further comments to make.

Is the work clearly and accurately presented and does it cite the current literature?

Partly

If applicable, is the statistical analysis and its interpretation appropriate?

Partly

Are all the source data underlying the results available to ensure full reproducibility?

Partly

Is the study design appropriate and is the work technically sound?

Partly

Are the conclusions drawn adequately supported by the results?

No

Are sufficient details of methods and analysis provided to allow replication by others?

No

Reviewer Expertise:

Obstetrics and Gynaecology

I confirm that I have read this submission and believe that I have an appropriate level of expertise to confirm that it is of an acceptable scientific standard.

# Reviewer report: Reviewer response for version 1

**DOI:** 10.5256/f1000research.181350.r392395

**Published:** 2025-06-30

**Authors:** Mathew Olumide Adebisi

**Affiliations:** 1 Obstetrics and Gynaecology, Federal Teaching Hospital Ido-Ekiti (Ringgold ID: 605037), Ido Ekiti, Ekiti, Nigeria; 2 Afe Babalola University, Ado-Ekiti, Ekiti, Nigeria

**GENERAL COMMENTS**

 The manuscript delved into an important obstetric mishap that is a major contributor to maternal morbidity and mortality especially in developing nations.

 However, manuscript needs major revisions especially in the areas of literature review on the existing knowledge and knowledge gap, methodology, result presentation, discussion and conclusion.

**SPECIFIC COMMENTS ON SECTIONS**

**Title:** Nothing remarkable and it is well written.

**Abstract:** “The aim is to identify factors associated with complete uterine rupture (UR)”. In your methodology, asymptomatic UR cases (complete/incomplete) were analyzed. This should be harmonized to make the methodology reproducible and thoroughly understood by the readers.

 Result (in the abstract section): ……. protective effect per kilogram decrease?

 This statement should be clearly mentioned. For example, …. protective effect per kilogram maternal body weight.

**Main text:**

 Introduction: This is grossly inadequate. Are there no studies on the topic of silent uterine rupture either scarred or unscarred from developing countries?  Are there no studies on how asymptomatic UR cases are being recognized either by imaging or at surgery?

 Suggested citations:

- Ebeigbe PN et.al. (2005) - **Ref. 1**
- Kadowa I. (2010) **- Ref. 2**
- Fofie C and Baffoe P (2011) - **Ref. 3**

There are still many more that can be cited to add to the literature review.

 Methodology: Is this study only on complete uterine rupture? Your stated in the methodology that “asymptomatic uterine rupture cases (complete/incomplete) were analyzed to compare clinical profiles, identify risk factors and assess maternal and neonatal outcomes. Also, result section revealed comparison of complete UR versus incomplete UR.

 You also stated that symptomatic cases and those in labour or other emergency settings were excluded from the study. However, some of the tables (2 and 4) in the result section show duration of labour as variable. I suggest that the different sections of the manuscript should be harmonized.

 Statistical analysis: Is this study a randomized study?

 Results:

 Parturient refers to a woman in labour and those in labour were said to be excluded.

 Other terms or phrases could be used to describe the 41 cases. For example; Among the 41 women with asymptomatic uterine rupture…..

 Tables 2 and 4- Harmonized the Methodology and the variables.

 Discussion:

 Quoting your statement from this section- “This cohort showed no demographic (Age, body mass index, socioeconomic status) or obstetric (quality of antenatal care, gestational diabetes) association with uterine rupture outcomes. However, it was reported that two “new predictors” emerged; namely (1) prolonged labour, leading to uterine rupture is a reflection of the type of **obstetric care** received (2) lower maternal weight.

 “Gestational age and delivery time were also noted to be longer in the incomplete uterine rupture cases”. These are also obstetric /labour factors.

 I therefore suggest that the exclusion criteria (and by extension, the methodology) and result presentation should be revised.

 Conclusion: The study reported that two new predictors emerged which is a strong point in this study. However, authors concluded on factor which the study did not reveal. The conclusion should be revised.

 References: some references have been suggested. If the literature review (introduction section) is revised as recommended, definitely 2 or more citations will be added to the reference.

Is the work clearly and accurately presented and does it cite the current literature?

Partly

If applicable, is the statistical analysis and its interpretation appropriate?

Partly

Are all the source data underlying the results available to ensure full reproducibility?

Partly

Is the study design appropriate and is the work technically sound?

Partly

Are the conclusions drawn adequately supported by the results?

No

Are sufficient details of methods and analysis provided to allow replication by others?

No

Reviewer Expertise:

Obstetrics and Gynaecology

I confirm that I have read this submission and believe that I have an appropriate level of expertise to confirm that it is of an acceptable scientific standard, however I have significant reservations, as outlined above.

## References

[1] : Ruptured uterus in a Nigerian community: a study of sociodemographic and obstetric risk factors. *Acta Obstetricia et Gynecologica Scandinavica* .2005;84(12) : 10.1111/j.0001-6349.2005.00778.x 1172-1174 10.1111/j.0001-6349.2005.00778.x 16305703

[2] : Ruptured uterus in rural Uganda: prevalence, predisposing factors and outcomes dust. *https://pubmed.ncbi.nlm.nih.gov/20200773/* .2010;20200773

[3] : A two-year review of uterine rupture in a regional hospital. *Ghana Medical Journal* .2011;44(3) : 10.4314/gmj.v44i3.68892 10.4314/gmj.v44i3.68892 PMC299683821327013

[4] : Silent Danger: Risk Factors and Outcomes of Fortuitously Discovered Uterine Rupture - ; A 41-Case Cohort Study. *https://dataverse.harvard.edu/dataset.xhtml?persistentId* .2025; 10.7910/DVN/D9OO16 10.7910/DVN/D9OO16 PMC1239512940893737

# Response

**Published:** 2025-08-02

**Authors:** Narjes karmous

**Affiliations:** University of Tunis El Manar Faculty of Medicine of Tunis, Tunis, Tunis, Tunisia

Dear Mathew Olumide Adebisi,

 We sincerely appreciate your constructive feedback. Your insightful comments have helped us refine and enhance the quality of our manuscript.

**Reviewer comment:**

**Abstract: Harmonize the aim and methodology regarding complete/incomplete UR.**

**Author response:**

 The Abstract has been revised to clearly state that both complete and incomplete forms of asymptomatic uterine rupture were included and analyzed in the study, ensuring coherence with the Methods and Results sections.

**Reviewer comment:**

**Result (Abstract): Clarify “protective effect per kilogram decrease”.**

**Author response:**

 We revised the phrasing in the Abstract to “a protective effect per kilogram of maternal body weight” for clarity and statistical accuracy.

**Reviewer comment:**

**Introduction is grossly inadequate. Literature on silent uterine rupture in developing countries should be reviewed. Suggested citations: Ref. 1–3.**

**Author response:**

 The Introduction has been substantially expanded and now includes data from studies conducted in Nigeria, Uganda, and Ghana, as suggested (references [7]–[9]). These studies help contextualize our findings within a broader regional framework of silent uterine rupture and its risk factors.

**Reviewer comment:**

**Methodology unclear: Complete vs incomplete UR? Harmonize with Results.**

**Author response:**

 This issue has been addressed. We now explicitly state in the Methods that both complete and incomplete asymptomatic uterine ruptures were included in the study and compared across multiple variables. Terminology and consistency have been corrected throughout.

**Reviewer comment:**

**Tables 2 and 4 mention labor variables, yet laboring women were excluded.**

**Author response:**

 We clarified that “asymptomatic” in our study refers to the absence of clinical signs of rupture (pain, bleeding, fetal distress), but not necessarily the absence of labor. Some patients had labor activity before cesarean delivery, which justifies the inclusion of labor variables. This is now clearly stated in the 'Study Population' and 'Discussion' sections.

**Reviewer comment:**

**Is this a randomized study?**

**Author response:**

 No. We confirm this is a retrospective observational cohort study. This has been clearly stated in the revised Methodology section.

**Reviewer comment:**

**Replace “parturient” with more appropriate term for asymptomatic cases.**

**Author response:**

 We have replaced the term “parturient” with “women with asymptomatic uterine rupture” or more specific language throughout the manuscript for precision and accuracy.

**Reviewer comment:**

**Discussion contradicts exclusion criteria: Prolonged labor and gestational age as predictors?**

**Author response:**

 This has been clarified. Although these women had no rupture symptoms, many experienced labor prior to cesarean. This distinction has been made clear in the revised Discussion and Methods sections.

**Reviewer comment:**

**Conclusion not supported by results. Revise accordingly.**

**Author response:**

 We have revised the Conclusion to focus strictly on statistically supported findings: prolonged labor and maternal weight <68 kg as independent predictors of complete rupture. Speculative elements were removed.

**Reviewer comment:**

**References: more literature needed if Introduction is revised.**

**Author response:**

 We have included all the suggested references and added several recent publications to strengthen the Introduction and Discussion.

**Reviewer comment:**

**Are sufficient details of methods and analysis provided to allow replication by others? → No**

**Author response:**

 The Methods section has been substantially revised to improve clarity and reproducibility. It now includes more precise definitions of study population, variables, criteria, and analysis thresholds.

**Reviewer comment:**

**Are the conclusions drawn adequately supported by the results? → No**

**Author response:**

 The conclusions have been adjusted to align fully with the results. We now only report findings that were statistically demonstrated and explicitly acknowledge study limitations.

 Sincerely,

 Narjes Karmous

# Reviewer report: Reviewer response for version 1

**DOI:** 10.5256/f1000research.181350.r392398

**Published:** 2025-06-25

**Authors:** Phuc Nhon Nguyen

**Affiliations:** 1 Tu Du Hospital, Ho Chi Minh City, Vietnam

Thank you for paper. I have some comments:

 In introduction, the study should mention some etiologies of uterine rupture, even in rare cases of placenta accreta spectrum on unscarred uterus. PMCID: PMC9568723

 The inclusion criteria: “At least one previous uterine surgery” is necessary?

 A study flowchart with inclusion/exclusion cases should be added.

 Maternal weight < 68kg is a protective factor? Could the authors please explain the relationship between maternal weight and uterine rupture? The neonatal weight may be more significant?

 Blood loss and blood transfusion should be presented additionally as median [IQR].  

 Definition of important variable should be added. For example: complete/incomplete UR.

 BMI should be represented instead of separate weight and height.

 The small sample size made the CI of multivariate logistic regression too large. This is confused.

 Please discuss why the period 2014-2017 related to increasing number of UR cases?

 In discussion, some other etiologies of intraabdominal hemorrhage in pregnancy should be also noted as well as role of imaging modalities such as ultrasound. doi: 10.1186/s12245-023-00498-w.

 Pulmonary embolism complication should be prevented in cesarean with severe blood loss.refer to 1

 Please discuss the role of cardiotocography in monitoring labor and detecting UR. refer to 2

 Change of fetal head's position during labor should be exanimated. 

 Practical points should be summarized.

Is the work clearly and accurately presented and does it cite the current literature?

Partly

If applicable, is the statistical analysis and its interpretation appropriate?

I cannot comment. A qualified statistician is required.

Are all the source data underlying the results available to ensure full reproducibility?

Partly

Is the study design appropriate and is the work technically sound?

Partly

Are the conclusions drawn adequately supported by the results?

Partly

Are sufficient details of methods and analysis provided to allow replication by others?

Partly

Reviewer Expertise:

Pregnancy pathology

I confirm that I have read this submission and believe that I have an appropriate level of expertise to confirm that it is of an acceptable scientific standard, however I have significant reservations, as outlined above.

## References

[1] : Successfully conservative management of the uterus in acute pulmonary embolism during cesarean section for placenta previa: a case report from Tu Du Hospital, Vietnam and literature review. *International Journal of Emergency Medicine* .2024;17(1) : 10.1186/s12245-024-00587-4 10.1186/s12245-024-00587-4 38287235 PMC10823749

[2] : Predicting the risks and recognizing the signs: a two-year prospective population-based study on pregnant women with uterine rupture in The Netherlands. *The Journal of Maternal-Fetal & Neonatal Medicine* .2024;37(1) : 10.1080/14767058.2024.2311083 10.1080/14767058.2024.2311083 38350236

# Response

**Published:** 2025-08-02

**Authors:** Narjes karmous

**Affiliations:** University of Tunis El Manar Faculty of Medicine of Tunis, Tunis, Tunis, Tunisia

Dear Phuc Nhon Nguyen,

 We sincerely appreciate your constructive feedback. Your insightful comments have helped us refine and enhance the quality of our manuscript.

**Reviewer comment: In introduction, the study should mention some etiologies of uterine rupture, even in rare cases of placenta accreta spectrum on unscarred uterus. PMCID: PMC9568723**

**Author response: **We appreciate this suggestion. A sentence addressing rare etiologies of uterine rupture, including placenta accreta spectrum in unscarred uteri, has been added to the Introduction, with citation [6].

**Reviewer comment: The inclusion criteria: “At least one previous uterine surgery” is necessary?**

**Author response: **Thank you for your insightful comment. We acknowledge that our earlier wording may have created some confusion. To clarify: a history of uterine surgery was not required for inclusion in this study. Our aim was to analyze all cases of fortuitously discovered uterine rupture, regardless of uterine surgical history.

 As such, the presence or absence of a uterine scar was not used as an inclusion criterion. What defined eligibility was the asymptomatic nature of the rupture and its intraoperative or immediate postpartum discovery, combined with complete medical documentation. Both scarred and unscarred uteri were represented in our cohort.

 We have updated the Materials and Methods section accordingly to ensure clarity and consistency with this inclusion approach.

**Reviewer comment: A study flowchart with inclusion/exclusion cases should be added.**

**Author response: **A flowchart has been included as Figure 1 to illustrate the inclusion and exclusion process clearly.

**Reviewer comment : Maternal weight < 68kg is a protective factor? Could the authors please explain the relationship between maternal weight and uterine rupture? The neonatal weight may be more significant?**

**Author response : **This point has been expanded in the Discussion, with added references [21–26] explaining the physiopathological links between obesity, uterine healing, and rupture. The role of neonatal weight is also discussed.

**Reviewer comment : Blood loss and blood transfusion should be presented additionally as median [IQR].**

**Author response: **We have revised Table 3 to present blood loss and transfusion data as median [IQR], in line with reviewer recommendations.

**Reviewer comment: **Definition of important variable should be added. For example: complete/incomplete UR.

**Author response: **Definitions of 'complete' and 'incomplete' uterine rupture have been added to the Methods section under the 'Definitions' subsection.

**Reviewer comment: BMI should be represented instead of separate weight and height.**

**Author response: **We replaced separate weight and height with BMI throughout the manuscript and added BMI statistics in Table 2.

**Reviewer comment: The small sample size made the CI of multivariate logistic regression too large. This is confused.**

**Author response: **We agree. This limitation is acknowledged explicitly in the Methods and Discussion sections.

**Reviewer comment: Please discuss why the period 2014–2017 related to increasing number of UR cases?**

**Author response: **We provided an explanation in the Discussion, linking the peak in 2017 to institutional practices and changes in intraoperative exploration and documentation.

**Reviewer comment: In discussion, some other etiologies of intraabdominal hemorrhage in pregnancy should be also noted as well as role of imaging modalities such as ultrasound.**

**Author response: **These aspects have been discussed in the revised Discussion, with references [17,18,19].

**Reviewer comment: Pulmonary embolism complication should be prevented in cesarean with severe blood loss.**

**Author response: **We agree. The importance of thromboprophylaxis has been added to the Discussion, citing reference [29].

**Reviewer comment: **Please discuss the role of cardiotocography in monitoring labor and detecting UR.

**Author response: **The role of CTG as an early indicator of uterine rupture has been included in the Discussion with appropriate reference [27].

**Reviewer comment: Change of fetal head's position during labor should be examined.**

**Author response:** This was mentioned in the Discussion as a potential indirect sign of uterine dehiscence, citing reference [28].

**Reviewer comment: Practical points should be summarized.**

**Author response: **We added a bullet-style summary of clinical implications and recommendations at the end of the Discussion section.

 Sincerely,

 Narjes Karmous
